# Prebiotic protein design supports a halophile origin of foldable proteins

**DOI:** 10.3389/fmicb.2013.00418

**Published:** 2014-01-06

**Authors:** Liam M. Longo, Michael Blaber

**Affiliations:** Department of Biomedical Sciences, College of Medicine, Florida State UniversityTallahassee, FL, USA

**Keywords:** halophile, abiogenesis, proteogenesis, prebiotic, protein folding

**Figure 1:**
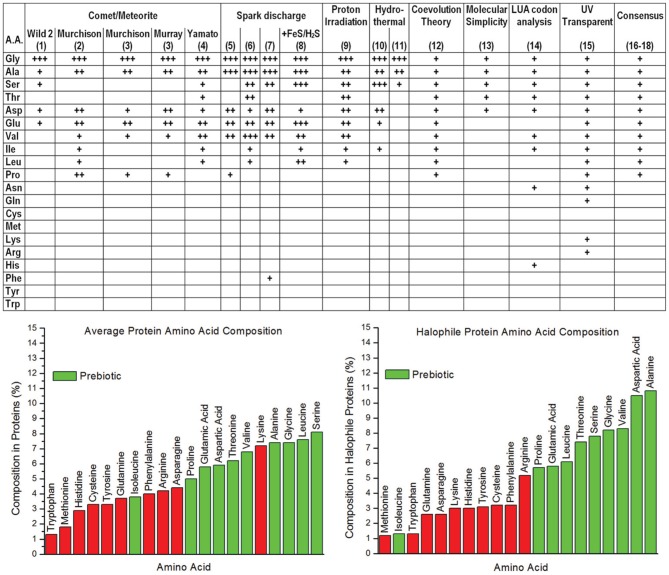
**Upper panel:** Consensus of prebiotic availability of the 20 common proteogenic α-amino acids from analysis of comets/meteorites, Miller Urey type spark discharge experiments, proton irradiation synthesis, hydrothermal vent synthesis, coevolution theory, molecular simplicity, last universal ancestor codon analysis, and UV transparency [for references of datasets (1–8), (10–11), and (16–18) see Longo and Blaber (2012); (9) Takano et al. (2004); (12) Wong (2005); (13) Cleaves (2010); (14) Brooks et al. (2002); (15) Cockell and Airo (2002)]. **Lower panel:** (left) the average α-amino acid composition in all proteins, with green indicating the prebiotic amino acids, Red bars indicate biotic amino acids (King and Jukes, 1969; Dyer, 1971); (right) the average α-amino acid composition in halophile proteins, with green indicating the prebiotic amino acids, Red bars indicate biotic amino acids (Fukuchi et al., 2003).

There are significant challenges in forming testable hypotheses regarding abiogenesis (i.e., the origin of life); for example, the original environment on the early Earth during the process of abiogenesis is a matter of debate [although it was significantly different from the current environment (Oparin, 1952; Hazen et al., 2008)]. Furthermore, the process of abiogenesis occurred over a time scale that is impractical to replicate as a laboratory experiment. More difficult still is the likelihood that current life forms are far removed from the earliest “living systems”—which may well have utilized entirely different initial energetic, biochemical, and “genetic” systems. Despite such difficulties, there are potentially testable hypotheses regarding the origin of *important classes* of biomolecules from abiotic processes. A key biomolecule that emerged in abiogenesis is the foldable polypeptide, which ultimately evolved to provide essentially all of the important biochemical and structural machinery in living systems. Each of the generally-acknowledged abiotic chemical processes present during abiogenesis, including atmospheric spark discharge, hydrothermal vent chemistry, as well as deep-space synthesis and delivery of organic material via comet and asteroid bombardment, can produce a subset of the 20 common α-amino acids [for a summary see Longo and Blaber (2012)]. Such prebiotic amino acids would have provided the raw material for the earliest polypeptides (i.e., “proteogenesis”); thus, the properties of such amino acids and polypeptides are of special interest. As with all things related to abiogenesis, the set of prebiotic amino acids available for proteogenesis has been a matter of debate; however, a compilation of broad and diverse analyses is arguably converging upon a consensus set of 10 prebiotic α-amino acids (Figure 1).

The alphabet size and chemical properties of the prebiotic α-amino acids are critical parameters as regards the capability to form foldable polypeptides. The rules of protein folding are not fully understood; however, some essential requirements of amino acids to promote folding are known. The tertiary structure of folded proteins is an assemblage of common secondary structure elements—including α-helix, β-strand, and reverse-turns. Thus, a foldable set of amino acids includes representatives with a high propensity to form each of the common secondary structure elements. Additionally, soluble globular proteins typically fold so as to sequester hydrophobic side chains within the protein interior, and this forms a significant energetic contribution to the overall stability of the folded protein; thus, a foldable set of amino acids contains both hydrophobic and hydrophilic members. Finally, functional considerations require that among the amino acids is a representative that can act as a nucleophile, and thereby provide useful chemical activity to an otherwise benign structural scaffold. While folding requirements are demanding, it is clear that the extant set of 20 common α-amino acids is redundant in this regard. Thus, the question of the *minimum* α-amino acid alphabet necessary to enable protein foldability has also been investigated, with a proposed minimum alphabet size of 9–10 amino acids (Romero et al., 1999; Murphy et al., 2000). Thus, with regard to set size, the prebiotic α-amino acid alphabet is located on the very cusp of foldable potential—a precarious position indeed, as it would provide essentially no redundancy in the requirements for protein foldability. Viewed in these terms, the prebiotic set is *remarkable* in containing all necessary elements for foldability—including high-propensity amino acids for each of the basic types of protein secondary structure, hydrophobic and hydrophilic amino acids, as well as several nucleophilic amino acids [for a discussion of such properties see Longo and Blaber (2012)]. However, the characteristics of a protein comprised of the prebiotic set of α-amino acids present a *stark deviation from the majority of extant proteins*, since aromatic residues, key contributors to the critical hydrophobic effect that drives protein folding, are absent in the prebiotic alphabet. Furthermore, there are no basic amino acids in the prebiotic set (McDonald and Storrie-Lombardi, 2010), thus restricting protein design to exclusively acidic polypeptides—limiting the presence of salt bridge interactions and resulting in acidic pI.

A number of successful studies of simplified protein design have been reported whereby foldable proteins have been constructed from a reduced α-amino acid alphabet, and relevance for proteogenesis have been described. However, such studies have focused exclusively upon achieving minimization of the *alphabet size*, without regard to the *prebiotic relevance* of the included amino acid alphabet. Thus, without exception, such minimal foldable proteins have depended upon critical aromatic amino acids within the core, as well as stabilizing salt bridges (dependent upon basic amino acids), to achieve a stable structure—no minimal protein design has utilized a plausible prebiotic alphabet. Thus, while minimal alphabets can yield foldable polypeptides, the foldable potential of the set of prebiotic amino acids has not been explored with the necessary rigor.

To determine the folding potential of the set of prebiotic α-amino acids our lab evaluated the consequences of enriching for the prebiotic set in a designed β-trefoil protein. The β-trefoil is a common protein architecture that has been the subject of much study as regards its evolutionary emergence from a simple 42-mer peptide motif (Lee and Blaber, 2011; Lee et al., 2011). Two “primitive” versions of the β-trefoil protein were subsequently designed; in primitive version 1 (PV1) the amino acid alphabet was reduced to 13 unique amino acids with an overall prebiotic composition of 74%; in primitive version 2 (PV2) the amino acid alphabet was reduced to 12 unique amino acids with an overall prebiotic composition of 79%. Notably, PV2 is devoid of any aromatic amino acids. The hydrophobic core of the β-trefoil architecture involves a substantial number of positions (21 total; or ~17% of total positions), and with PV2 this important region of the protein is *entirely prebiotic* and comprised of only 3 different amino acids (Leu, Ile, and Val). In addition to reduced core hydrophobicity the PV1 and PV2 proteins exhibit a substantial increase in acidic property (due to the exclusively acidic nature of the prebiotic set of amino acids). A high negative surface charge density is a characteristic feature of halophilic proteins, enabling them to remain soluble in high salt via carboxylate binding of solvated metal cations. Additionally, halophile proteins are characterized as having reduced hydrophobicity, and denaturation under low salt conditions. This property of prebiotic amino acid composition and compatibility with a high salt environment is understood principally in terms of the biophysics of protein solubility in high salt enabled by surface acidic charge that binds hydrated salt cation and the effect of salt in stabilizing the hydrophobic effect. In low salt buffer PV2 is only fractionally folded even at its temperature of maximum stability. However, high salt stabilizes the PV2 protein, shifting its melting temperature into the region of high-mesophile/low-thermophile stability and exhibiting >99% fractional native state; thus, by the criteria of efficient foldability PV2 is an *obligate halophilic protein* (Longo et al., 2013). While high salt also stabilizes PV1, it is not essential for folding stability as the aromatic residues in the core result in efficient hydrophobic packing within the β-trefoil architecture. The crystal structure of the PV2 protein showed a substantial acidic surface charge characteristic of halophile proteins, and distinctly different from the initial mesophile β-trefoil protein from which PV2 was derived. Thus, by several defining criteria the enrichment of prebiotic amino acids in creating the PV2 protein had produced a halophile protein (Longo et al., 2013). The PV2 protein, however, is not 100% prebiotic in its amino acid composition, and further work to achieve an entirely prebiotic foldable protein is needed to support the hypotheses put forth in this opinion article.

A reasonable postulate of abiogenesis is that some residual aspect of the process may still be identifiable in extant organisms. Protein machinery in extant organisms can be profoundly complex—as can be seen in molecular assemblies such as ATPase, ribosomes, cilia, the proteasome, pyruvate dehydrogenase complex, myosin, and others; however, such complex protein assemblages are built up from *remarkably simple α-amino acids that can be synthesized by abiotic chemical processes*. The amino acid composition of proteins is enriched for the prebiotic set, with 64% of amino acids being prebiotic (Figure 1); however, the composition of halophile proteins shows a substantially greater enrichment (72%) of prebiotic amino acids (Figure 1). Thus, it is compelling to speculate that this signature is a legacy of abiogenesis—in that *the properties of the halophile environment are highly compatible with foldable polypeptides derived from available prebiotic α-amino acids*. The halophile environment is typically thought of as a curious niche that mesophiles adapted into; however, it has also been proposed as an appropriate environment for abiogenesis and proteogenesis (Dundas, 1998; Rode, 1999). Studies of the folding potential of the set of prebiotic α-amino acids suggest that the halophile environment was a potentially ideal cradle for the proteogenic process in abiogenesis.
